# Overall Survival of Patients With Unresectable or Metastatic BRAF V600-Mutant Acral/Cutaneous Melanoma Administered Dabrafenib Plus Trametinib: Long-Term Follow-Up of a Multicenter, Single-Arm Phase IIa Trial

**DOI:** 10.3389/fonc.2021.720044

**Published:** 2021-08-24

**Authors:** Lili Mao, Ya Ding, Xue Bai, Xinan Sheng, Jie Dai, Zhihong Chi, Chuanliang Cui, Yan Kong, Yun Fan, Yanjun Xu, Xuan Wang, Bixia Tang, Bin Lian, Xieqiao Yan, Siming Li, Li Zhou, Xiaoting Wei, Caili Li, Jun Guo, Xiaoshi Zhang, Lu Si

**Affiliations:** ^1^Key Laboratory of Carcinogenesis and Translational Research (Ministry of Education/Beijing), Department of Melanoma, Peking University Cancer Hospital & Institute, Beijing, China; ^2^Department of Biotherapy, State Key Laboratory of Oncology in South China, Collaborative Innovation Center for Cancer Medicine, Sun Yat-Sen University Cancer Center, Guangzhou, China; ^3^Key Laboratory of Carcinogenesis and Translational Research (Ministry of Education/Beijing), Department of Genitourinary Oncology, Peking University Cancer Hospital & Institute, Beijing, China; ^4^Cancer Hospital of the University of Chinese Academy of Sciences (Zhejiang Cancer Hospital), Institute of Cancer and Basic Medicine (IBMC), Chinese Academy of Sciences, Hangzhou, China

**Keywords:** melanoma, dabrafenib, trametinib, BRAF, acral melanoma

## Abstract

**Objectives:**

To examine the long-term survival outcome of dabrafenib in combination with trametinib in Chinese patients with unresectable or metastatic acral/cutaneous melanoma with BRAF-V600 mutation and to explore potential predictors of effectiveness.

**Methods:**

This was a long-term follow-up of Chinese patients with unresectable or metastatic BRAF V600-mutant acral/cutaneous melanoma administered dabrafenib (150 mg twice daily) plus trametinib (2 mg once daily) in an open-label, multicenter, single-arm, phase IIa study (NCT02083354). Efficacy endpoints included objective response rate (ORR), duration of response (DOR), progression-free survival (PFS), and overall survival (OS). The impacts of baseline characteristics on PFS and OS were analyzed.

**Results:**

A total of sixty patients were included. The median age was 48 years, and 24 patients (40.0%) were male. Totally 12 individuals (20.0%) had acral melanoma, and 45 (75.0%) had failed previous systemic therapy. Up to July 2020, the median duration of follow-up was 37.0 (95% confidence interval [CI] 29.1-44.9) months. The updated ORR was 71.7% (95%CI 60.3%-83.1%). The 3-year OS rate was 28.8% (95%CI 19.1-43.6%) in the overall population, and 35.7% (95%CI 15.5–82.4%) in acral melanoma patients. The median DOR was 7.5 months (95%CI 4.5 to 10.5). Baseline normal lactic dehydrogenase (LDH), metastatic organ sites<3 and complete response to combination therapy with dabrafenib plus trametinib were associated with improved PFS and OS.

**Conclusion:**

Dabrafenib combined with trametinib confer long-term survival in Chinese patients with BRAF V600-mutant, unresectable or metastatic acral/cutaneous melanoma.

**Clinical Trial Registration:**

https://clinicaltrials.gov/ct2/show/NCT02083354, identifier NCT02083354.

## Introduction

Although the incidence of melanoma is only 0.9 per 100,000 persons in China, it has been increasing for the past 20 years (1). Most melanoma cases are in locally advanced stage at the time of diagnosis, with some eventually developing metastatic disease, which results in poor prognosis (2, 3). BRAF mutations play a critical role in melanoma initiation and progression, and about 25% of all Chinese melanoma patients harbor BRAF mutations (4). BRAF mutation rate varies by anatomic type. Indeed, BRAF mutations were reported in 50% cutaneous melanoma and 15% acral melanoma cases; the latter is the most common subtype in Chinese melanoma patients (3, 4).

Major strides have been made in the treatment of advanced melanoma with BRAF mutation in recent years. Compared with chemotherapy, BRAF inhibitors have significantly improved the survival of BRAF mutant patients (5, 6). In addition, the combination of BRAF and MEK inhibitors circumvents the drug resistance caused by mitogen-activated protein kinase (MAPK) pathway reactivation when BRAF inhibitors are used alone, without increasing the overall toxicity (7–11). Although PD-1 blockade was effective regardless of the patient’s BRAF mutation status in some trials (12, 13), existing data demonstrated an ORR of 15% in Chinese BRAF V600-mutant melanoma patients treated with pembrolizumab (14).

A recent phase IIa trial evaluated the efficacy and safety of dabrafenib plus trametinib in 77 East Asian patients with unresectable or metastatic BRAF V600-mutant cutaneous melanoma, including 61 (79.2%) from China’s mainland. The preliminary results of this trial demonstrated an ORR of 61.0% and a median progression-free survival (PFS) of 7.9 months (15). However, the median follow-up time was 8.3 months in the above report at the time of data cutoff (February 23, 2018), and median overall survival (OS) was not reached due to the relatively short follow-up. Therefore, overall survival in Chinese patients with unresectable or metastatic BRAF V600-mutant melanoma administered the dabrafenib plus trametinib regimen remains unknown.

Here, we report updated the ORR, PFS and OS of patients treated with dabrafenib plus trametinib in this phase IIa study in China’s mainland, including the data of acral melanoma cases. We also provide an analysis of factors that might be associated with derived long-term benefit from this combination therapy.

## Methods

### Study Design and Participants

The original study was an open-label, multicenter, single-arm, phase IIa trial (ClinicalTrials.gov, NCT02083354) conducted in East Asia (China’s mainland, Hong Kong, Taiwan, Korea, and Thailand) (15). Chinese mainland melanoma patients, treated in Peking University Cancer Hospital, Sun Yat-Sen University Cancer Hospital, and Zhejiang Cancer Hospital between March 2014 and November 2017, were included in the present analysis.

Briefly, eligible patients were ≥18 years of age, with histopathologically confirmed stage IIIc (unresectable) or IV (metastatic) melanoma, BRAF V600 mutation according to the central reference laboratory, at least one measurable lesion according to Response Evaluation Criteria in Solid Tumors (RECIST) version 1.1 (16), Eastern Cooperative Oncology Group (ECOG) performance status of 0 or 1, and adequate organ function. Individuals with primary mucosal or ocular melanoma were excluded. The study was conducted in accordance with the Declaration of Helsinki and Good Clinical Practice, and approved by the ethics committee of each participating center. Written informed consent was obtained from each patient.

### Treatment

Patients received dabrafenib at 150 mg twice daily plus trametinib at 2 mg once daily by oral administration until disease progression (PD), death, unacceptable toxicity, withdrawal of consent, or discontinuation for any reason. Dose modifications were allowed for the management of adverse events (AEs).

### Endpoints

Patients were followed up every 28 days. The primary endpoint was ORR. Tumor response was assessed by an investigator according to RECIST 1.1 at baseline, every 8 weeks until week 56, and then every 12 weeks until disease progression or death. Secondary endpoints included PFS, OS, and the duration of response (DOR). Post-hoc defined endpoints included time to response (TTR) and post progression survival (PPS). AEs were recorded and graded according to the National Cancer Institute Common Terminology Criteria for Adverse Events, version 4.0, during the study period (17).

### Statistical Analysis

Statistical analysis was performed with SPSS 22.0 (IBM, Armonk, NY, USA) or GraphPad Prism version 8.0 (GraphPad Software). The Clopper–Pearson method was used to calculate the 95% confidence interval (CI) of the ORR. ORRs were compared by the chi-square test. Progression-free survival and overall survival were estimated by the Kaplan–Meier method, and compared by the log-rank test. P<0.05 was considered statistically significant.

## Results

### Baseline Characteristics of the Participants

A total of 61 patients were enrolled in this study, and one withdrew consent before treatment. In the 60 patients included in this analysis, the median age was 48 years (range, 26-76 years), and 24 (40.0%) individuals were male. Twelve (20.0%) patients had melanomas at acral sites. Thirty-seven (61.7%) exhibited normal lactic dehydrogenase (LDH) amounts. Forty-one (68.3%) had visceral disease, including 14 (23.3%) with liver metastasis and 4 (6.7%) with central nervous system metastases. Thirty-five (58.3%) patients had ≥3 organ sites with metastasis at baseline. Fifteen cases (25.0%) were treatment-naïve, while forty-five (75.0%) received at least one line of therapy. Nine (15.0%) patients had prior immunotherapy including PD-1 inhibitor with or without CTLA-4 inhibitor (**Table 1**).

**Table 1:** Baseline characteristics of the participants.

Characteristics	Number (%)
Median age (range)	48.0 (26.0 - 76.0)
Male	24 (40.0)
ECOG performance status	
0	20 (33.3)
1	40 (66.7)
Primary site	
Acral	12 (20.0)
Trunk	32 (53.3)
Limb	4 (6.7)
Head & Neck	5 (8.3)
Unknown	7 (11.7)
Tumor stage at screening	
Unresectable stage IIIc	2 (3.3)
Stage IV	
M1a	10 (16.7)
M1b	14 (23.3)
M1c	31 (51.7)
Unknown	3 (5.0)
LDH	
≤ULN	37 (61.7)
>ULN	23 (38.3)
Lines of previous therapies	
0	15 (25.0)
1	19 (31.7)
2	19 (31.7)
3	6 (10.0)
4	1 (1.7)
Previous immunotherapy	
PD-1 inhibitor only	6 (10.0)
CTLA-4 inhibitor only	2 (3.3)
Both	1 (1.7)
Number of organ sites with metastasis	
1	12 (20.0)
2	13 (21.7)
≥3	35 (58.3)

Data are number (%), unless otherwise indicated. ECOG, Eastern Cooperative Oncology Group; CSD, chronically sun-damaged cutaneous melanoma; LDH, lactic dehydrogenase; ULN, upper limit of normal; PD-1, programmed cell death protein-1; CTLA-4, cytotoxic T lymphocyte associate protein-4.

### Efficacy

CR, PR and stable disease (SD) were achieved in 5 (8.3%), 38 (63.3%) and 17 (28.3%) patients, respectively. Overall, the ORR was 71.7% (43/60). Among the 15 treatment-naïve patients examined, the objective response rate was 86.7%. In patients with ≥1 previous therapy (n=45), the objective response rate was 66.7%. The response rates were 83.3% in the acral melanoma subgroup and 70.8% in the non-acral subgroup. Notably, acral melanomas also exhibited high response to combination therapy (**Table 2**). There was no significant difference in ORR among patients with different lines of prior therapy, primary sites, LDH levels or metastatic organ sites (**Table S1 in the Supplementary Appendix**).

**Table 2:** Tumor response and ORR by subgroups.

Response	Number (%)
Type of response	
Complete response (n=60)	5 (8.3)
Partial response (n=60)	38 (63.3)
Stable disease (n=60)	17 (28.3)
Progression disease	0
Objective response rate (n=60)	43 (71.7)
Previous therapy	
Naïve (n=15)	13 (86.7)
Treated (n=45)	30 (66.7)
Primary site	
Acral (n=12)	9 (83.3)
Non-acral (n=48)	34 (70.8)
LDH	
Normal (n=37)	27 (73.0)
Elevated (n=23)	16 (69.6)
Organ sites	
<3 (n=25)	21 (84.0)
≥3 (n=35)	22 (62.9)

Data are number (%), unless otherwise indicated. LDH, lactic dehydrogenase.

**Figure 1:**
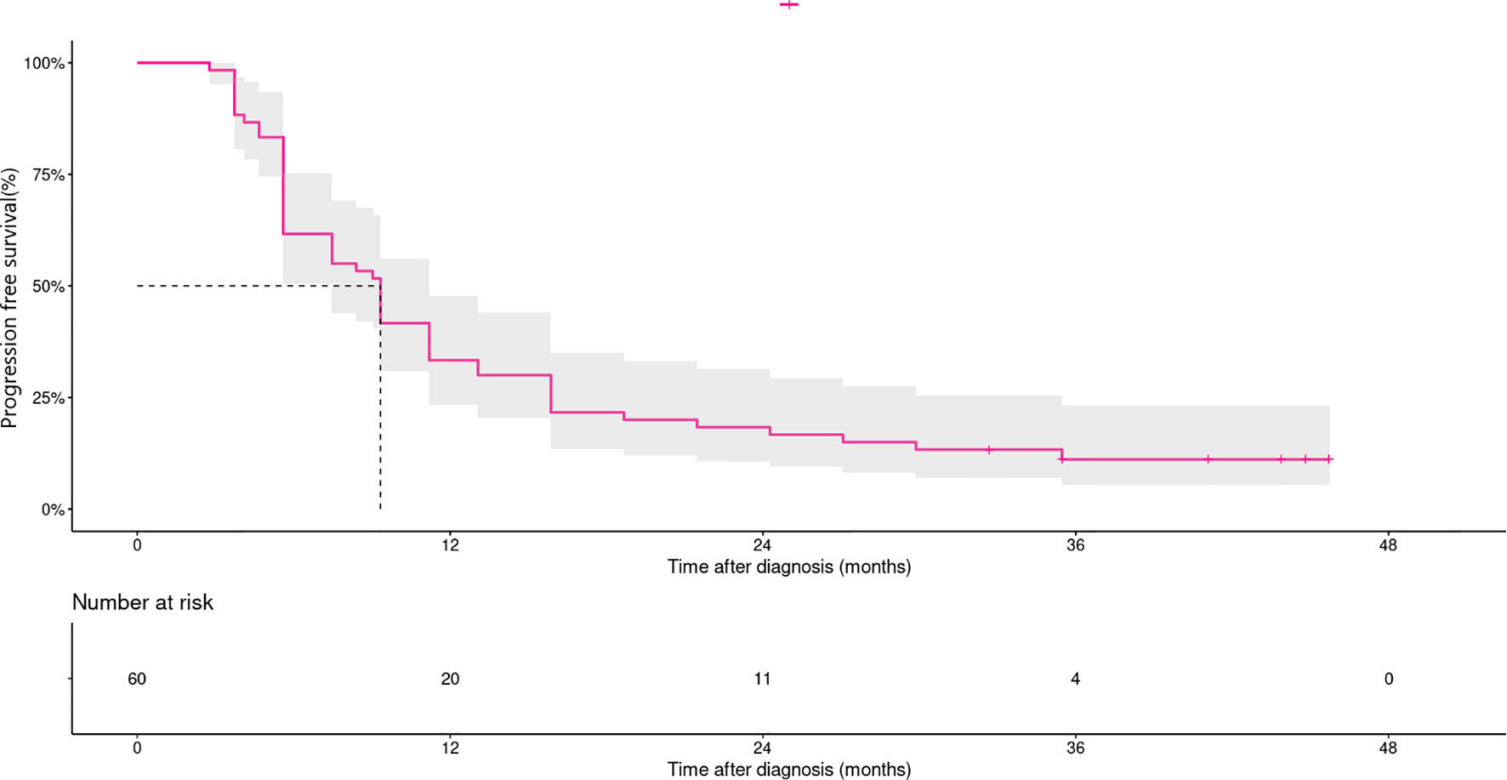
Kaplan–Meier curves for progression-free survival in patients treated with dabrafenib and trametinib (n=60). Median PFS and 3 year-PFS rates were 9.3 month (95% CI, 8.4–10.3) and 11.1% (95% CI, 3.3–18.9%), respectively.

At data cut-off, the median follow-up time was 37.0 months (95%CI 29.1–44.9). In the overall population, median PFS and 3 year-PFS rates were 9.3 month (95%CI 8.4–10.3) and 11.1% (95%CI 3.3–18.9%), respectively (**Figure 1**). Three year-PFS rates were 20.0% (95%CI 7.3–55.0%) and 8.9% (95%CI 3.5-22.7) in treatment-naïve and pretreated patients, respectively. Ten of the 12 patients with acral melanoma progressed in 2 years, and maximum PFS time was less than the landmark timepoint. Patients with non-acral melanoma had a 3 year-PFS rate of 10% (95%CI 4.2–23.8%). Patients with normal baseline lactate dehydrogenase levels had a 3-year PFS rate of 18.9% (95%CI 9.7-36.9%), *versus* 0% in patients with elevated lactate dehydrogenase levels. Patients with metastatic organ sites <3 had a 3-year PFS rate of 24% (95%CI 11.9-48.2%), *versus* 2.9% (95%CI 4 to 19.7%) in patients with ≥3 sites (**Figure S1 in the Supplementary Appendix**).

**Figure 2:**
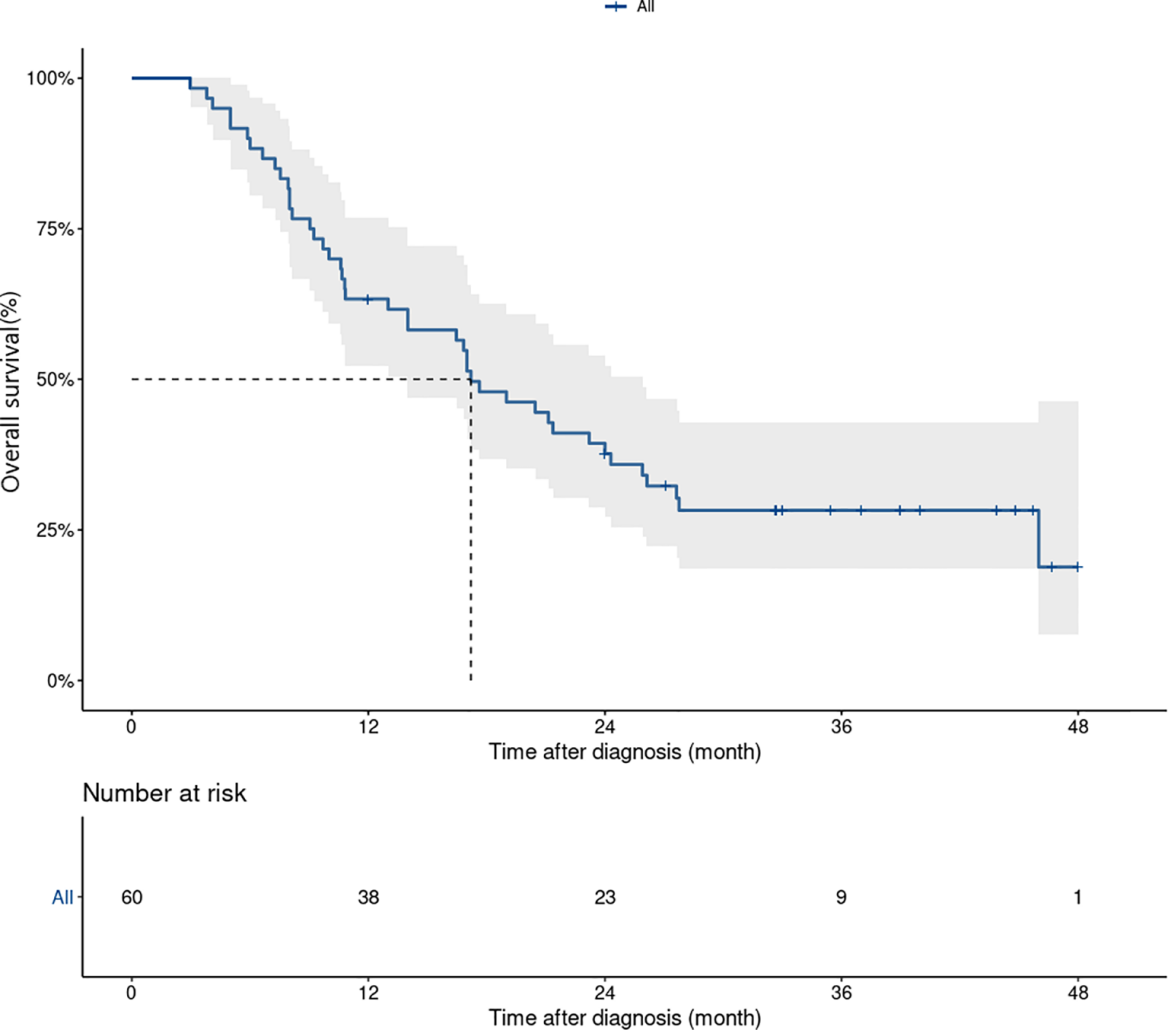
Kaplan–Meier curves for overall survival in patients treated with dabrafenib and trametinib (n=60). Median OS and 3 years OS rate were 17.6 (95% CI, 13.1–22.1) month and 28.8% (95% CI, 19.1-43.6%), respectively.

Median OS and 3-year OS rate were 17.6 (95%CI 13.1–22.1) months and 28.8% (95%CI 19.1-43.6%), respectively (**Figure 2**). Three year-OS rate was 44.5% (95%CI 23.2-85.4%) in treatment-naïve patients, *versus* 24.2% (95%CI 14.4-40.7%) in pretreated patients. In patients with acral melanoma and non-acral melanoma, 3-year OS rates were 35.7% (95%CI 15.5–82.4%) and 27% (95%CI 16.8–43.7%), respectively. Patients with normal baseline lactate dehydrogenase levels had a 3-year OS rate of 40.8% (95%CI 27.3-61.1%), *versus* 9.3% (95%CI 2.5-34.8%) in those with elevated lactate dehydrogenase levels at baseline. Patients with metastatic organ sites<3 had a 3-year OS rate of 53.1% (95%CI 36.0-78.1%), *versus* 11.9% (95%CI 4.8-29.9%) in those with ≥ 3 sites (**Figure S2 in the Supplementary Appendix**).

There were no significant differences in PFS and OS among subgroups based on lines of previous therapies or primary sites. Normal LDH levels and metastatic organ sites<3 were associated with improved PFS and OS (**Figures S1** **and** **S2 in the Supplementary Appendix**).

**Figure 3:**
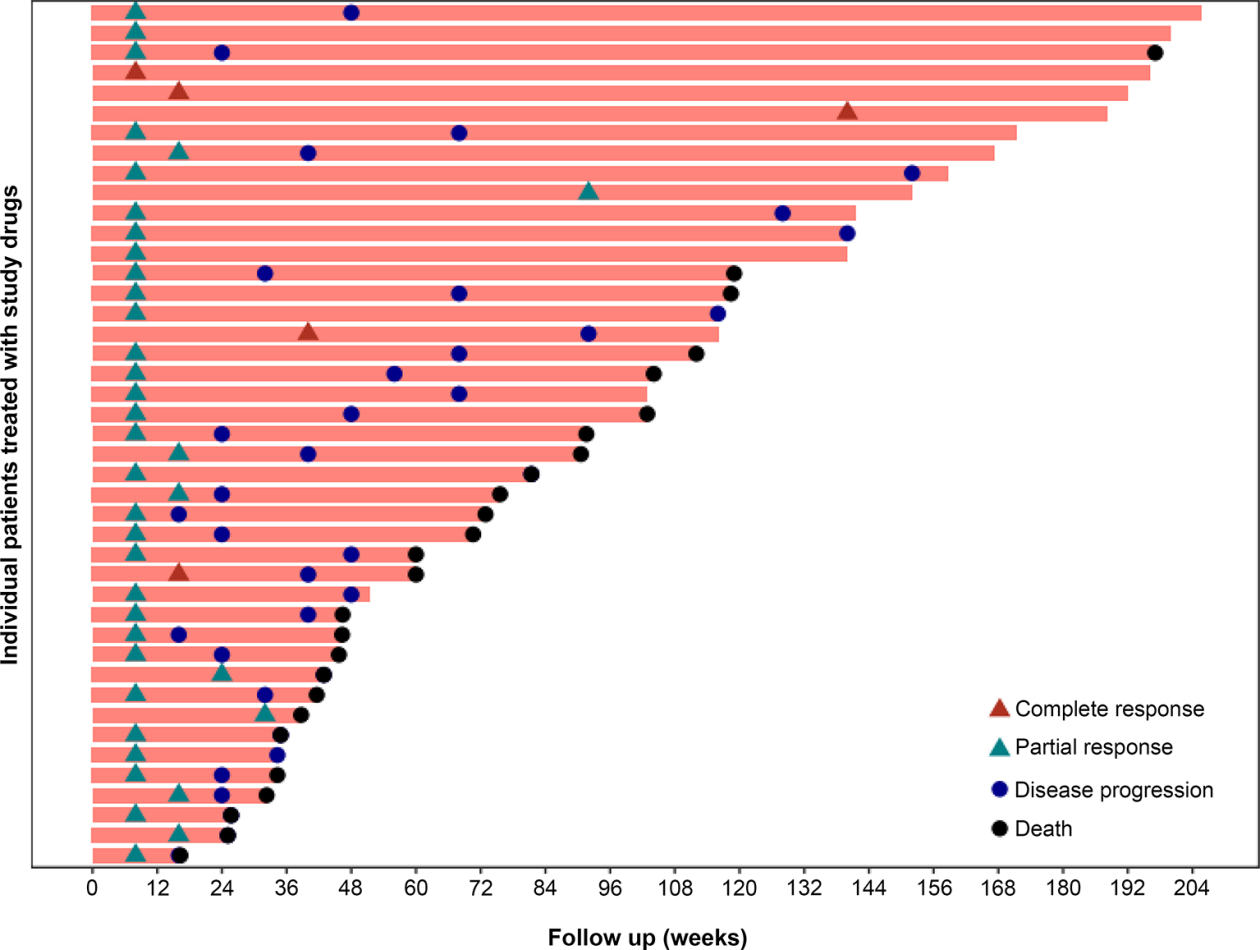
Time to response and duration of study treatment. A total of 43 of the 60 patients had a response, including 5 patients with CR and 38 patients with PR. In the 43 patients with best response of CR or PR, the median TTR was 1.9 months.

The median DOR was 7.5 months (95%CI 4.5-10.5) in the overall population. In patients with best response of CR or PR, the median TTR was 1.9 months. **Figure 3** presents the DOR of each patient who achieved CR or PR.

### Post-Treatment and Survival After Disease Progression

At the time of this analysis, a total of 53 events of disease progression were noted. Median survival after disease progression in the overall population was 7.1 months (95%CI 4.2–10.0). Twenty-four of the 53 (45.2%) patients received subsequent anti-tumor therapies, including chemotherapy (12/53, 22.6%), immune checkpoint inhibitors (14/53, 26.4%), angiogenesis inhibitors (14/53, 26.4%), BRAF with or without MEK inhibitor re-challenge (8/53, 15.1%), and radiation therapy (2/53, 3.8%) (**Table S3 in the Supplementary Appendix**). Patients (n=10) administered a PD-1 inhibitor after disease progression post-treatment with dabrafenib plus trametinib achieved a median post progression survival (PPS) of 17.6 months (95%CI 16.9–28.3) (**Figure S3 in the Supplementary Appendix**). Eight patients received re-challenge therapy with BRAF with or without MEK inhibitors; one achieved PR and six had tumor shrinkage, of whom three were alive until the last follow-up (median PPS of 40.4 months).

### Characteristics of the Patients Who Achieved CR or OS >3 Years

Five patients achieved CR, including 2 females and 3 males; four patients had normal LDH levels (126–164 U/L), and one had elevated LDH (330 U/L). Two patients were treatment naïve, and 3 were chemotherapy-treated. None of these patients underwent immunotherapy previously. All the 5 patients were M1a/b, and only 1 had visceral disease (lung); median baseline tumor size (BTS) was 20 mm (range, 15-34 mm; BTS was quantified as the sum of the longest dimensions of all measurable baseline target lesions). The detailed data of individuals who achieved CR are shown in **Table S4 in the Supplementary Appendix**. Patients who achieved CR had better survival (PFS, not available (NA); OS, NA) compared with those who achieved PR [PFS, 9.3 months (95%CI 8.9–9.8); OS, 21.4 months (95%CI 13.8–28.9)] or SD [PFS, 5.6 months (95%CI 4.3–6.9); OS, 10.6 months (95%CI 3.8–17.4)] (P<0.05) (**Figure S4 in the Supplementary Appendix**).

A total of 9 patients had OS beyond 3 years. Their median age was 42 years (range, 29-66), and they included 4 males. Eight patients had normal LDH, 1 had primary melanoma located in the acral area, 7 had metastatic organ sites<3, and four had previous immunotherapy before enrollment. Three male patients achieved complete response, and 6 patients achieved a partial response as best overall response. At the time of this analysis, 3 male patients were still on treatment, and four patients received subsequent anti-tumor therapies, of whom two achieved CR after subsequent chemotherapy. **Table S4 in the Supplementary Appendix** shows the detailed features of patients with OS >3 years.

### Adverse Events

Treatment-related AE occurred in 53 patients (53/60, 88.3%). The most common AEs were pyrexia (31/60, 51.7%), anemia (25/60, 41.7%) and neutropenia (23/60, 38.3%). Ten of the sixty (13.3%) patients had dose reduction because of treatment-related AEs, including hypertension (one patient), elevated ALT/AST (one patient), and fever/chills (eight patients). Four of the sixty (6.7%) cases discontinued treatment due to treatment-related AEs, including pigment epithelial detachment (one patient), interstitial lung disease (one patient), fever/chills (one patient), and ejection fraction decrease (one patient). With an additional 28 months of follow-up since the last analysis, no new safety issues were reported.

## Discussion

This 3-year analysis showed that in Chinese patients with unresectable or metastatic BRAF V600-mutant melanoma, median OS and 3-year OS rate were 17.6 months and 23%, respectively, after treatment with dabrafenib plus trametinib. These data, which represent the longest follow-up of dabrafenib plus trametinib in Chinese melanoma patients to date, confirm the durable antitumor activity and safety of D+T in advanced and metastatic acral/cutaneous melanoma.

The safety profile of D+T in patients with melanoma has been established partly in a previous report (15); with continued follow-up, no new safety signals have been identified in this study.

Notably, 3-year PFS and OS rates in patients administered first-line treatment with dabrafenib plus trametinib were 22% and 44%, respectively. These results were consistent to 3-year landmark analysis results observed in a randomized, double-blinded, phase III Combi-D trial (18), which compared the combination of dabrafenib and trametinib to dabrafenib monotherapy as first-line therapy in patients with unresectable or metastatic BRAF V600E/K mutation-positive cutaneous melanoma. These data suggested that Chinese patients have similar clinical benefit as the Caucasian counterparts after treatment with first line dabrafenib and trametinib combination therapy for metastatic melanoma. There was a trend towards better ORR or survival outcomes in patients administered first line treatment compared with pretreated patients, although there was no significant difference.

Acral melanoma is the most common subtype in the Chinese population (19, 20), with lower incidence of BRAF mutations than cutaneous melanoma (4). In this study, 12 acral melanoma patients (20%) were enrolled. Acral melanoma is considered to be more aggressive with worse prognosis compared with cutaneous melanoma (21, 22). It is thought to be largely resistant to immunotherapy (14, 20, 23, 24). The present analysis demonstrated that acral melanoma patients could achieve favorable ORR, although median PFS and median OS were slightly lower in the acral subgroup compared with non-acral cases; 3-year PFS and OS rates in patients with acral melanoma were similar to those of cutaneous melanoma cases. Therefore, the BRAFi+MEKi combination might be the preferred strategy for the acral melanoma subtype with BRAF V600 mutation.

Multivariate analysis of baseline factors demonstrated that LDH levels and the number of metastatic sites were significantly associated with PFS and OS, corroborating previous randomized trials in Caucasian melanoma patients (9, 25, 26).

A recently published retrospective study demonstrated the clinical efficacy and safety of a combination therapy consisting of BRAF and MEK inhibitors (dabrafenib plus trametinib) in Japanese patients with unresectable or metastatic BRAF V600-mutant cutaneous melanoma (27). Of note, these patients had an ORR of 72.3%, a median PFS of 12 months and a median OS of 23 months. These findings confirmed that melanoma patients could achieve a favorable response and durable survival benefit with dabrafenib plus trametinib in East-Asian patients.

Regarding secondary endpoints, median PFS (9.3 months) achieved with dabrafenib and trametinib in this trial was similar to survival outcomes of the phase II BRF113220 trial (9, 28), as well as the phase III Combi-D trial with combination-targeted therapy (7). While median OS (17.6 months) seemed shorter than in the abovementioned trials (25.0 months), the differences in post-trial therapies were limited.

In the five-year outcome analysis of COMBI-v and COMBI-d trials evaluating 563 patients administered dabrafenib plus trametinib, CR occurred in 109 patients (19%) and was associated with improved long-term outcome, with an OS rate of 71% (95%CI 62 to 79) at 5 years (29). In the five cases who achieved CR in this study, lines of previous therapies were no more than two, no patients had prior immunotherapy, and metastatic organ sites were all under three. The four patients without visceral disease had longer PFS and OS, and neither death nor disease progression occurred in three of them. Therefore, patients with fewer lines of previous therapy, no previous immunotherapy, fewer metastatic organ sites and no visceral disease might gain more survival benefits from the combined regimen. Furthermore, while analyzing survival in patients with different tumor response in this trial, optimal outcomes were observed in individuals who achieved CR, confirming previous analyses (25, 26).

Nine cases had overall survival longer than 3 years, of whom 5 were progression free at the time of analysis. Longer OS may be attributed to longer PFS, fewer previous therapies, small baseline tumor size, less metastatic organ sites, and better response.

After disease progression post-treatment with dabrafenib plus trametinib, post progression survival (PPS) in the whole population was 7.1 months. Ten patients who were switched to PD-1 inhibitors achieved a PPS of 17.6 months, while BRAF re-challenge also conferred clinical benefit to a subgroup of patients. The further treatment choice for progression after combination therapy needs further investigation for confirmation. Overall survival may be affected by subsequent treatment after administration of dabrafenib plus trametinib; however, without strict response assessment and data collection beyond the clinical-trial setting, the correlation between PPS and OS remains unclear. Furthermore, there is an unmet need for optimal treatments to overcome resistance to BRAF and MEK inhibitors. Given the breakthrough of immunotherapy in melanoma, immune checkpoint inhibitors combined with targeted drugs might be an option for selected patients (28–30).

The present study had some limitations. First, this was a subgroup analysis of a phase IIa trial (15). The sample size of this study might be inadequate, and the subgroup analysis was underpowered. Secondly, no control group was set for direct comparisons. Thirdly, the original study classified patients according to histological subtype (i.e., three patients had acral lentiginous melanoma) (15), while in the present study, patients were classified according to the involved primary site (i.e., 12 patients had acral melanoma). The discrepant numbers of patients with acral melanoma in both studies could be ascribed to different classification methods. Nevertheless, dabrafenib plus trametinib demonstrated preliminary clinical benefit in acral melanoma cases with BRAF V600 mutations. Large-scale clinical trials of dabrafenib plus trametinib with multicenter design are required to substantiate the present findings. As the effect of dabrafenib plus trametinib in mucosal melanoma is unknown, it deserves further investigation.

In conclusion, this analysis confirms the durable and robust antitumor activity and safety of dabrafenib combined with trametinib in Chinese patients with BRAF V600 mutation-positive melanoma, including acral melanoma patients. The efficacy of dabrafenib plus trametinib in a Chinese population was favorable, corroborating previous studies in Caucasian populations.

## Data Availability Statement

The raw data supporting the conclusions of this article will be made available by the authors, without undue reservation.

## Ethics Statement

The studies involving human participants were reviewed and approved by Ethics Committee of Beijing Cancer Hospital. The patients/participants provided their written informed consent to participate in this study.

## Author Contributions

JG and LS conceived of the presented idea. XZ, YD, YF, and YX contributed to data acquisition. LM and XB conducted the analyses and wrote the paper. The remaining authors contributed to collecting the data and finalizing this paper. LS is the guarantor for the article who accepts full responsibility for the work and/or the conduct of the study, had access to the data, and oversaw the decision to publish. All authors contributed to the article and approved the submitted version.

## Funding

The current work was supported by GlaxoSmithKline (GSK) in 2014. Dabrafenib and trametinib are assets of Novartis AG as of March 2, 2015. The study sponsorship has been transferred from GSK to Novartis in all participating countries except China. The funder was not involved in the study design, collection, analysis, interpretation of data, the writing of this article or the decision to submit it for publication.

## Conflict of Interest

LS has received speaker honoraria from MSD, Roche, Novartis, Shanghai Junshi Biosciences and OrienGene. Xiaoshi Zhang has consulting roles in MSD, Roche, Novartis, Shanghai Junshi. JG has consulting or advisory roles in MSD, Roche, Pfizer, Bayer, Novartis, Simcere Pharmaceutical Group, Shanghai Junshi Biosciences and OrienGene.

The remaining authors declare that the research was conducted in the absence of any commercial or financial relationships that could be construed as a potential conflict of interest.

## Publisher’s Note

All claims expressed in this article are solely those of the authors and do not necessarily represent those of their affiliated organizations, or those of the publisher, the editors and the reviewers. Any product that may be evaluated in this article, or claim that may be made by its manufacturer, is not guaranteed or endorsed by the publisher.
