# Supplementary material for: Overall Survival of Patients With Unresectable or Metastatic BRAF V600-Mutant Acral/Cutaneous Melanoma Administered Dabrafenib Plus Trametinib: Long-Term Follow-Up of a Multicenter, Single-Arm Phase IIa Trial
Source: Front Oncol. 2021 Aug 24;11:720044. doi: 10.3389/fonc.2021.720044 (PMC8422804; doi:10.3389/fonc.2021.720044)
Supplement: Supplementary file 1 [file DataSheet_1.docx]

**Table S1.** ORR in subgroups

| Subgroups | ORR, n (%) [95%CI] | P value |
| --- | --- | --- |
| ECOG performance status |  | 0.189 |
| 0 (n=20) | 14 (70.0) [49.9, 90.1] |  |
| 1 (n=40) | 29 (72.5) [58.7, 86.3] |  |
| Primary site |  |  |
| Cutaneous (n=39)^*^ | 25 (64.1) [49.2, 79.2] |  |
| Non-CSD (n=24) | 14 (58.3) [38.6, 78.0] |  |
| CSD (n=15) | 11 (73.3) [50.9, 95.7] |  |
| Acral (n=12) | 10 (83.3) [62.2, 100] |  |
| Unknown (n=9) | 8 (88.9) [68.4, 100] |  |
| LDH |  | 0.776 |
| ≤ULN (n=37) | 27 (73.0) [58.7, 87.3] |  |
| >ULN (n=23) | 16 (69.6) [50.8, 88.4] |  |
| Lines of previous therapies |  | 0.137 |
| 0 (n=15) | 13 (86.7) [69.5, 100] |  |
| 1 (n=19) | 13 (68.4) [47.5, 89.3] |  |
| ≥2 (n=26) | 17 (65.4) [47.1, 83.7] |  |
| Previous therapy |  |  |
| Chemotherapy (n=43) | 28 (65.1) [50.9, 79.3] |  |
| PD-1 inhibitor (n=7) | 6 (85.7) [59.8, 100] |  |
| other |  |  |
| Number of organ sites with metastasis |  | 0.07 |
| 1 (n=12) | 11 (91.7) [76.1, 100] |  |
| 2 (n=13) | 10 (76.9) [54.0, 99.8] |  |
| ≥3 (n=35) | 22 (62.9) [46.9, 78.9] |  |

*P=0.296, Cutaneous melanoma vs. acral melanoma.

ORR, objective response rate; CI, confidence interval; ECOG, Eastern Cooperative Oncology Group; CSD, chronically sun-damaged cutaneous melanoma; LDH, lactic dehydrogenase; ULN, upper limit of normal; PD-1, programmed cell death protein-1.

**Table S2.** Subsequent therapy after disease progression on dabrafenib plus trametinib

| Therapy | Patients with post-trial therapy (n =53) |
| --- | --- |
| Chemotherapy | 12 (22.6) |
| PD-1 inhibitor | 10 (18.9) |
| Apatinib | 6 (11.3) |
| Vemurafenib | 5 (9.4) |
| Bevacizumab | 4 (7.5) |
| Ipilimumab | 4 (7.5) |
| Anlotinib | 2 (3.8) |
| Continuing dabrafenib plus trametinib | 3 (5.7) |
| Endostar | 2 (3.8) |
| Radiation therapy | 2 (3.8) |
| Sorafenib | 2 (3.8) |
| Cobimetinib | 1 (1.9) |
| None | 25 (47.2) |

**Table S3.** Characteristics of patients who achieved CR

| Patients | Age (years) | Sex | ECOG performance status | Primary site | Tumor stage at screening | LDH (U/L) | Lines of previous therapies | BTS | Previous immunotherapy | Visceral disease | Number of organ sites with metastasis | PFS | OS |
| --- | --- | --- | --- | --- | --- | --- | --- | --- | --- | --- | --- | --- | --- |
| 1 | 36 | Male | 1 | Left calf | T2bN3M1a | 126 | 1 | 34 | No | No | 1 | 45.73 | 45.73 |
| 2 | 43 | Female | 0 | External auditory meatus | TxNxM1b | 330 | 1 | 17 | No | Yes | 2 | 11.00* | 14.0* |
| 3 | 26 | Female | 0 | Chest wall | TxNxM1a | 152 | 0 | 39 | No | No | 1 | 21.47* | 27.07 |
| 4 | 29 | Male | 1 | Left iliac fossa | cTxN3M1 | 164 | 0 | 15 | No | No | 3 | 44.80 | 44.80 |
| 5 | 65 | Male | 1 | Unknown | TxNxM1a | 156 | 2 | 20 | No | No | 1 | 43.87 | 43.87 |

* Patient experienced progression or death.

**Table S4.** Characteristics of patients with OS>3 years

| Patients | Age (years) | Sex | ECOG performance status | Primary site | Tumor stage at screening | LDH (U/L) | Lines of previous therapies | Previous immunotherapy | Visceral disease | Number of organ sites with metastasis | PFS | OS |
| --- | --- | --- | --- | --- | --- | --- | --- | --- | --- | --- | --- | --- |
| 1 | 31 | Male | 1 | Unknown | TxNxM1c | 164 | 3 | No | No | 1 | 11.2* | 48 |
| 2 | 55 | Female | 1 | Unknown | TxNxM1a | 167 | 1 | No | No | 1 | 41.07 | 46.67 |
| 3 | 47 | Female | 1 | Unknown | TxNxM1a | 187 | 0 | No | Yes | 2 | 5.6* | 46* |
| 4 | 36 | Male | 1 | Left calf | T2bN3M1a | 126 | 1 | No | No | 1 | 41.07 | 45.73 |
| 5 | 29 | Male | 1 | Left iliac fossa | cTxN3M1 | 164 | 0 | No | No | 3 | 44.8 | 44.8 |
| 6 | 65 | Male | 1 | Unknown | TxNxM1a | 156 | 2 | No | No | 1 | 43.87 | 43.87 |
| 7 | 42 | Female | 0 | Unknown | TxN3M1c | 153 | 1 | No | Yes | 1 | 15.87* | 40 |
| 8 | 32 | Female | 1 | right abdomen | T4aN1M1b | 418 | 3 | Yes | Yes | 3 | 9.33* | 39 |
| 9 | 61 | Female | 1 | right tempus | TxNxM1a | 139 | 3 | Yes | No | 2 | 35.47 | 37 |

**Figure legends**

**Figure S1** Progression-free survival comparison between (A) groups of first line and pretreated, (B)acral and non-acral, (C)normal LDH and elevated LDH, (D) metastatic organ sites<3 and ≥3

**Figure S2** Overall survival comparison between (A) first line and pretreated, (B)acral and non-acral, (C)normal LDH and elevated LDH, (D) metastatic organ sites<3 and ≥3

**Figure S3** post progression survival (PPS) in patients receiving anti-PD-1 therapy after dabrafenib and trametinib

**Figure S4** (A) progression-free survival and (B) overall survival by RECIST response. CR, complete response; NE, not estimable; OS, overall survival; PD, progressive disease; PFS, progression-free survival; PR, partial response; RECIST, Response Evaluation Criteria In Solid Tumors; SD, stable disease.
