# Supplementary figures and images for: Overall Survival of Patients With Unresectable or Metastatic BRAF V600-Mutant Acral/Cutaneous Melanoma Administered Dabrafenib Plus Trametinib: Long-Term Follow-Up of a Multicenter, Single-Arm Phase IIa Trial
Source: Front Oncol. 2021 Aug 24;11:720044. doi: 10.3389/fonc.2021.720044 (PMC8422804; doi:10.3389/fonc.2021.720044)

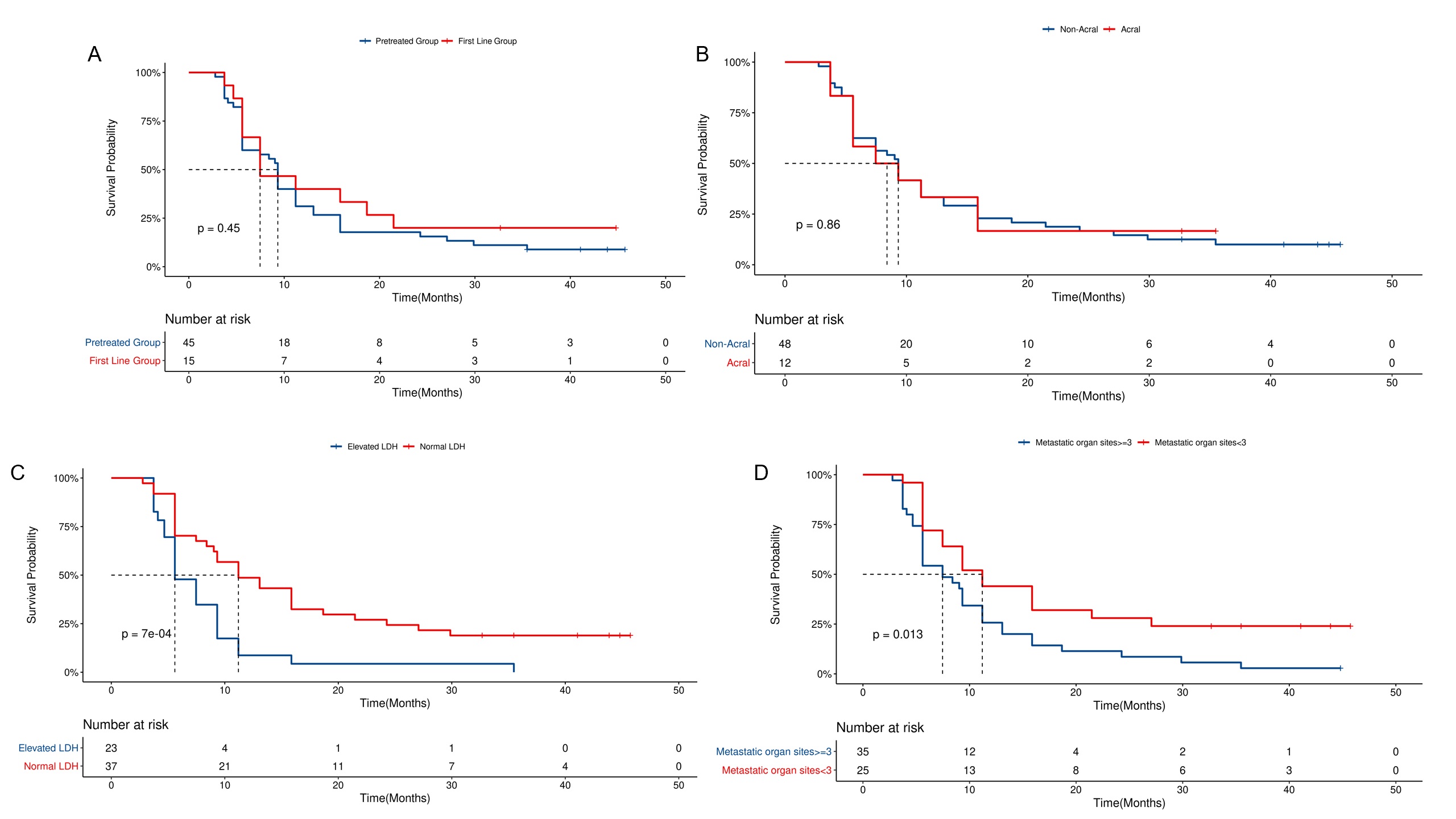

Supplement: Supplementary file 2 [file Image_1.jpg]

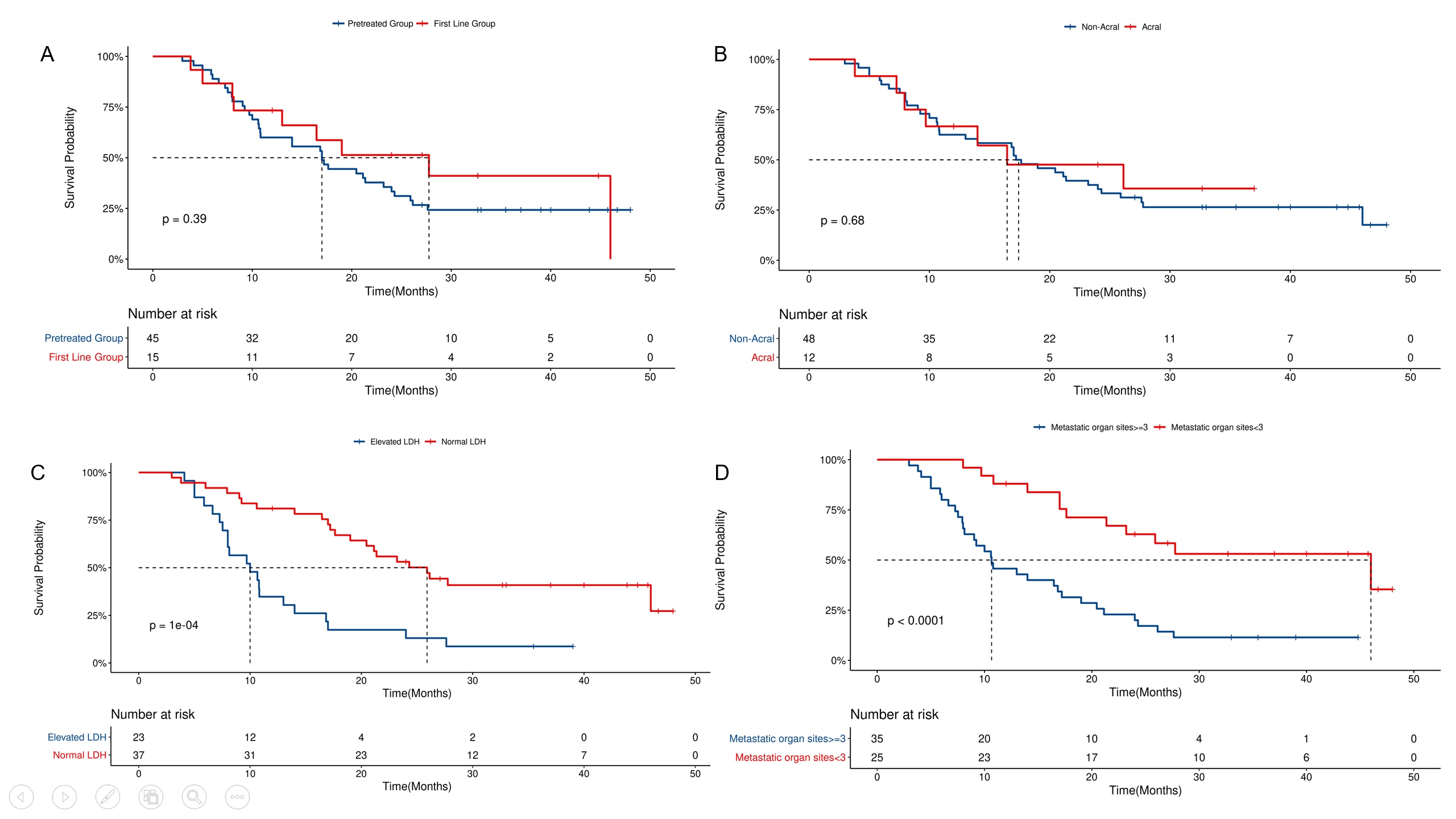

Supplement: Supplementary file 3 [file Image_2.jpg]

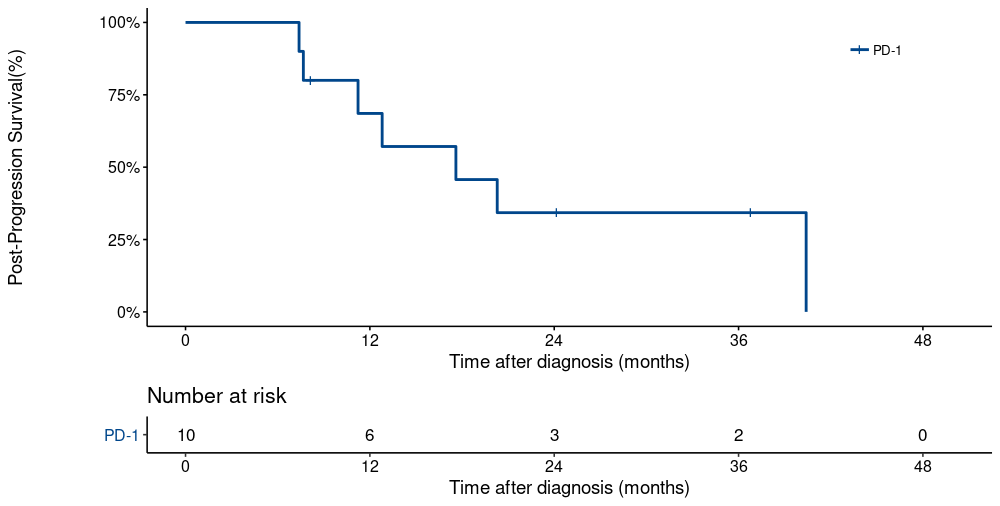

Supplement: Supplementary file 4 [file Image_3.tiff]

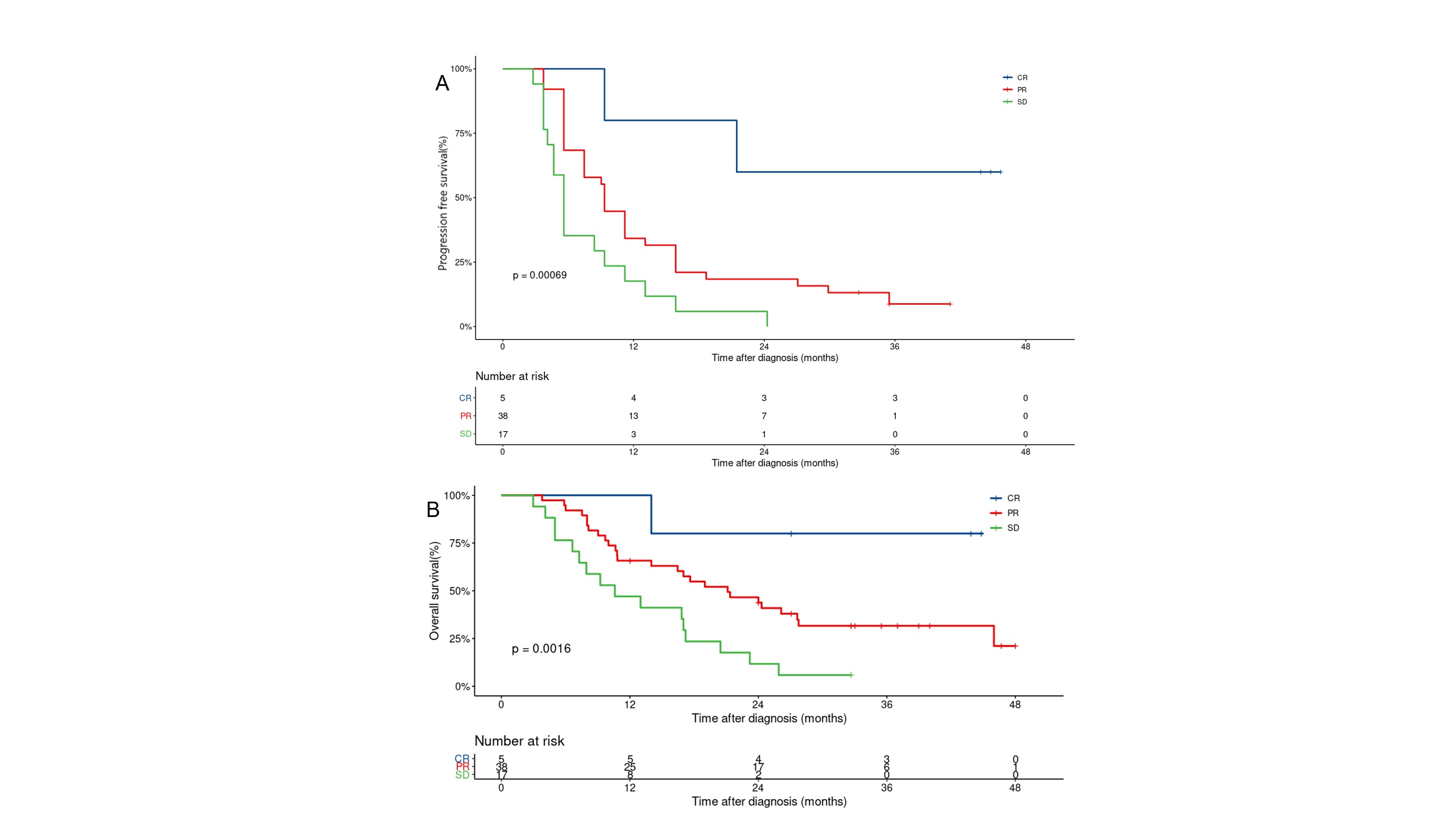

Supplement: Supplementary file 5 [file Image_4.jpg]
